# Fast Differential Analysis of Propolis Using Surface Desorption Atmospheric Pressure Chemical Ionization Mass Spectrometry

**DOI:** 10.1155/2015/176475

**Published:** 2015-08-03

**Authors:** Xue-yong Huang, Xia-li Guo, Huo-lin Luo, Xiao-wei Fang, Teng-gao Zhu, Xing-lei Zhang, Huan-wen Chen, Li-ping Luo

**Affiliations:** ^1^School of Life Sciences, Nanchang University, Nanchang, Jiangxi 330031, China; ^2^Jiangxi Key Laboratory for Mass Spectrometry and Instrumentation, East China Institute of Technology, Nanchang, Jiangxi 330013, China

## Abstract

Mass spectral fingerprints of 24 raw propolis samples, including 23 from China and one from the United States, were directly obtained using surface desorption atmospheric pressure chemical ionization mass spectrometry (SDAPCI-MS) without sample pretreatment. Under the optimized experimental conditions, the most abundant signals were detected in the mass ranges of 70 to 500 *m/z* and 200 to 350 *m/z*, respectively. Principal component analyses (PCA) for the two mass ranges showed similarities in that the colors had a significant correlation with the first two PCs; in contrast there was no correlation with the climatic zones from which the samples originated. Analytes such as chrysin, pinocembrin, and quercetin were detected and identified using multiple stage mass spectrometry within 3 min. Therefore, SDAPCI-MS can be used for rapid and reliable high-throughput analysis of propolis.

## 1. Introduction

Propolis, a natural resinous substance collected by honeybees from leaf buds and cracks in the bark of various plants, is thought to be used by the bees as a protective barrier against their enemies [1, 2]. Propolis has a complex chemical composition and exhibits antibacterial, antioxidant, antifungal, and antiviral properties [3–5]. The colors of propolis can be quite diverse, including yellow, black, yellow-green, and greenish-black. Crude propolis samples generally consist of 50% resin, 30% wax, 10% essential oils, 5% pollen, and 5% of various organic compounds [1]. More than 300 constituents have been identified in propolis [6–9]. The chemical composition and bioactivity of propolis are variable and mainly depend on the climate, the season, geographic characteristics, and the local flora exploited by bees [10, 11].

The main functions of propolis are attributed to key chemical components such as flavonoids, phenolic acids, and their esters. Since these lipophilic compounds are readily extracted by alcohol, recent studies and applications on propolis have mainly focused on ethanol extracts of propolis (EEP). There has also been much work on water extracts of propolis (WEP) and its volatile oils. The methods used for analysis and discrimination of propolis include HPLC [10, 12, 13], HPLC-ESI-MS [14], GC-MS [15, 16], LC-MS [17], and DHS-GC-O-MS [18]. Most of the above methods are time-consuming, laborious, and expensive and involve a considerable amount of manual work. Furthermore, the use of different sample preparation and analysis methods leads to a relative lack of standardization in methodology and noncomparability of results [19]. It is important, therefore, to develop a rapid, direct, and reliable procedure, capable of characterizing propolis samples in terms of a chemical fingerprint.

SDAPCI combines the processes of surface desorption and atmospheric pressure chemical ionization, two well-established techniques. SDAPCI can be operated without sheath gas and thus can be used for the analysis of powdered samples [20]. It has the merits of being nondestructive and offers high sensitivity and high throughput for detection of diverse compounds in complex matrices [21, 22] without sample pretreatment [23, 24]. SDAPCI-MS has been used to characterize diverse samples, including foods [21, 22], drugs [23], plants [23], and animal tissues [25, 26]. Propolis is rich in volatile components and secondary metabolites (polyphenols and terpenoids, etc.), molecular weights of which are generally less than 500. Therefore, we proposed that SDAPCI-MS may be suitable for the chemical analysis of propolis.

Recently, propolis fingerprints have been obtained using techniques such as HPLC, ESI-MS, EI-MS, and EASI-MS, for sample characterization and for determination of plant origin [27–31]. In this study, the MS fingerprints of 24 propolis samples, including 23 from China and one from the United States, were acquired by SDAPCI-MS as well as by HPLC, SDE-GC-MS, DHS-GC-MS, and DHS-GC-MS-O. We found that the fingerprints of Chinese propolis had significant correlation with their colors, but not with the climatic zones for the areas sampled. Then, we evaluated the applicability of SDAPCI-MS fingerprinting with principal component analysis (PCA) for direct, fast, and reliable characterization of crude propolis. To the best of our knowledge, there has been no similar such study published on SDAPCI-MS fingerprinting of crude propolis.

## 2. Materials and Methods

### 2.1. Instruments, Samples, and Reagents

A modified SDAPCI source built in our laboratory was interfaced to a commercial linear ion trap mass spectrometer (LTQ-XL, Finnigan, San Jose, CA, USA) and operated in the negative ion mode for direct analysis of propolis.

A total of 24 propolis samples were collected by beekeepers or ourselves, 23 being from 17 provinces in China and one from Illinois, USA. The collection areas, climate zones, and colors are shown in Table 1.

Quercetin was from the National Pharmaceutical Engineering Center for Solid Preparation in Chinese Herbal Medicine (Jiangxi, China). Chrysin was from the National Institute for the Control of Pharmaceutical and Biological Products (Beijing, China). Pinocembrin was from Sigma-Aldrich Chemicals Co., Ltd. (MO, USA).

### 2.2. Instrumental Setup

**Figure 1:**
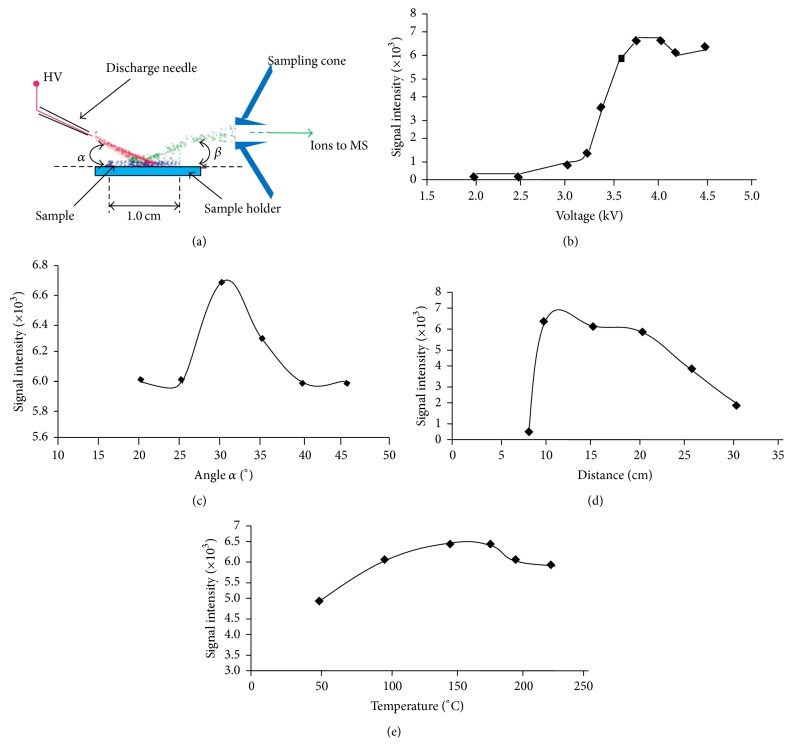
Schematic diagram of SDAPCI source and optimization of SDAPCI source conditions. (a) Schematic diagram of SDAPCI source for measurement of propolis. (b)–(e) Optimization of SDAPCI source conditions including the effect of discharge voltage on signal intensity (b), the effect of the angles *α* (c), the effect of the distance between the discharge tip and the ion entrance (d), and the effect of the temperature of the heated capillary (e). All the optimization experiments were based on the signal intensity of the peak signal at 121* m/z* for sample 1 (HLJ). Each point represents an average of six measurements.

A schematic diagram of the SDAPCI source is shown in Figure 1(a). The principle and the experimental setup for the SDAPCI have been described previously [32]. A cylindrical electrode with a cone on one end was secured by an insulator of 5 mm length exposed to the air. The LTQ-MS system was set for negative ion mode detection and the mass scan range was 65–1000* m/z*; the voltage of the discharge needle electrode was 3.5 kV, and the temperature of the capillary of the LTQ instrument was maintained at 275°C; the parent ions of interest were selected with a mass-to-charge window of 1.4 units; the collision-induced dissociation (CID) experiments were performed with 10–30 units of collision energy (CE) and 30 ms duration; all of the full-scan mass spectra were collected with an average time of 1 min and with background subtraction. Other parameters were optimized automatically by the LTQ-MS system. The distance between the discharge needle tip and the ion entrance was 10 mm and the distance between the discharge needle tip and the sample surface was 2 mm. The angle between the discharge needle and the sample surface was 30°, and the angle formed by the ion entrance capillary and the sample holder was 25°.

### 2.3. Procedures

#### 2.3.1. SDAPCI-MS Analysis

A thin, even layer (about 1 mm in thickness and 1 cm^2^ in size) of crude propolis powder was dispensed on a piece of filter paper, which was then placed on the sample holder under the discharge needle of the SDAPCI source. Water present in ambient air can be used directly as the reagent to generate the primary ions. The primary ions were accelerated by the electric field created by the high voltage on the needle, which then impacted on the sample surface. After momentum charge transfer and the extraction of analytes from the sample surface, a plume of droplets formed above the sample where analyte ions were then produced.

#### 2.3.2. Chemometric Analysis of Data

PCA was carried out by SPSS 18.01 for Windows (SASS, Chicago, Illinois, USA). For all samples, the spectra 70–500* m/z* and 200–350* m/z*, respectively, were used for PCA.

## 3. Results and Discussion

### 3.1. Optimization of SDAPCI Conditions for Crude Propolis Detection

The SDAPCI conditions were optimized by evaluating the signal intensities for propolis at 121* m/z*, as this ion was one of the most intense signals for most of the propolis samples in initial MS experiments (see supplementary Figure  1 in Supplementary Material available online at http://dx.doi.org/10.1155/2015/176475). As shown in Figure 1(b), signal intensity increased with the discharge voltage and reached its maximal level at 3.8 kV.  Figure 1(c) shows the signal was dependent on the angle *α*, and the maximal signal intensity was obtained at 30°.  Figure 1(d) shows that a distance of 1.0 mm was selected for the distance between the discharge tip and the ion entrance. The temperature of the capillary was optimized at 150°C, as shown in Figure 1(e). Under these optimized conditions, a measurement time of only 3 min, on average, was required for one sample.

### 3.2. Principal Component Analysis (PCA)

#### 3.2.1. PCA of the Spectra between 70 and 500* m/z*

PCA was carried out on the mass spectra between 70 and 500* m/z* (examples are shown in Supplementary Figure  1),  and 67.09% of the variance was explained by the three selected factors. PC1 accounted for 52.54% of the total variation, together with 10.13% for PC2 and 4.42% for PC3. The score values for each PC are shown in Table 2, as well as the loading values for the most abundant ions in all the samples. These ions were 78, 92, 93, 94, 102, 121, 151, 154, and 183* m/z*. Since crude propolis is rich in volatile components, these small molecules exhibited intense signals, which were readily detected. Up to now, these compounds have not been identified so it would be of interest to gain this new information.

**Figure 2:**
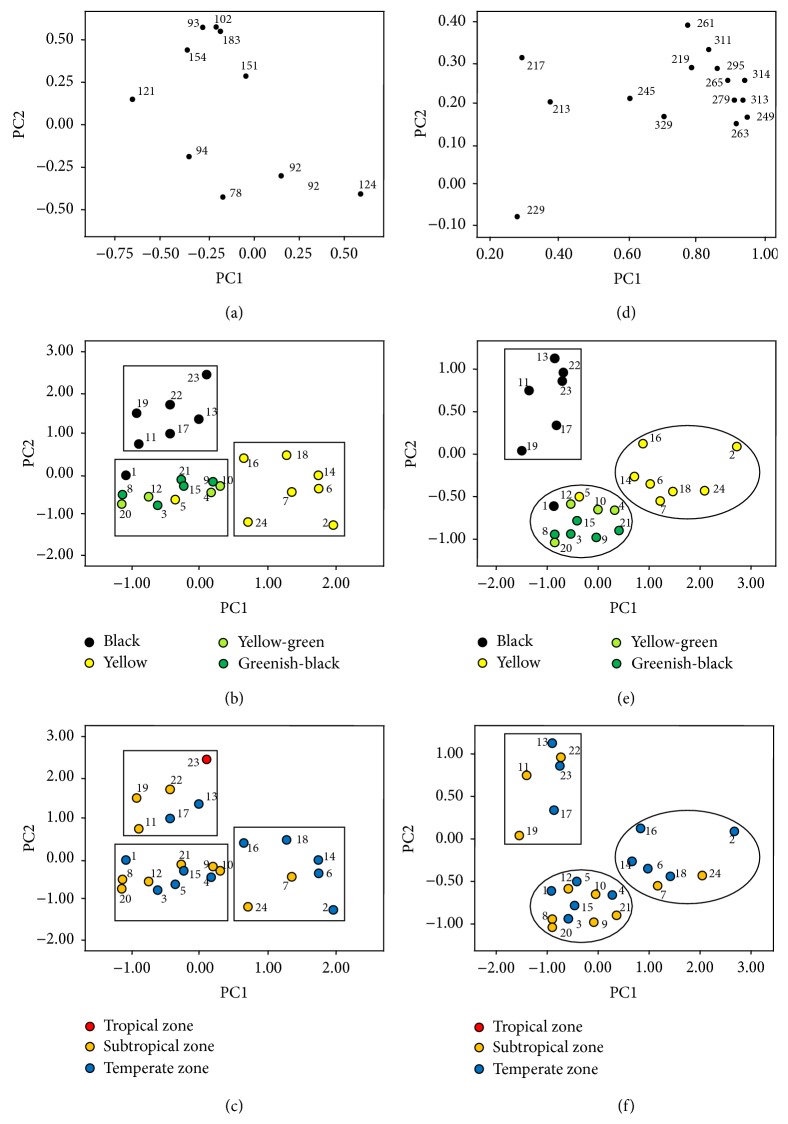
PCA analysis of SDAPCI-MS fingerprints. PCA loading plots (ions) for 70–500* m/z* (a) and 200–350* m/z* (d). PCA score plots for SDAPCI-MS fingerprints of propolis and their colors for 70–500* m/z* (b) and 200–350* m/z* (e). PCA score plots and their climatic zones for 70–500* m/z* (c) and 200–350* m/z* (f). For (a) and (d), the numbers of spots mean the* m/z* of the ions. For (b), (c), (e), and (f), the numbers are their sample number. See Table 1 for characteristics of the samples.

Figure 2(a) shows the associations between each of the abundant ions (78, 92, 93, 94, 102, 121, 151, 154, and 183* m/z*) and the first two PCs. Clearly, the propolis samples were classified into three groups. Group 1 contained sample numbers 11, 13, 17, 19, 22, and 23; group 2 contained samples 2, 6, 7, 14, 16, 18, and 24; and the rest formed group 3 (Figure 2(b)). As shown by our previous studies, the color of propolis was significantly associated with its quality significantly [12]. Therefore, we tried to reveal the relation between the fingerprints and the colors of Chinese propolis. We noted that most black propolis samples, except sample 1 (HLJ), belonged to group 1; and most yellow propolis samples, except sample 5 (HB-2), belonged to group 2; the other samples, including all the greenish-black and the yellow-green propolis samples belonged to group 3 (Figure 2(b)). These results suggested a significant correlation between the color of propolis samples and PC1 and PC2. A similar correlation was not observed between the climatic zone from which the propolis samples originated and the first two PCs (Figure 2(c)).

#### 3.2.2. PCA of the Spectra between 200 and 350* m/z*

We also carried out PCA based on the spectra between 200 and 350* m/z* (examples are shown in Supplementary Figure  2), and 80.50% of the variance was explained by the 3 selected factors. PC1 accounted for 44.71%, PC2 accounted for 22.77%, and PC3 accounted for 13.02% of the total variation. The score values for each PC are shown in Table 3, as well as loading values for the most abundant ions in all samples.

As shown in Table 3, the most abundant ions were 213, 217, 219, 229, 245, 249, 261, 263, 265, 279, 295, 311, 313, 314, and 329* m/z*. Other less abundant ions for fingerprinting of propolis, 247 (dihydroxyflavone), 253 (chrysin), 255 (pinocembrin), 267 (tectochrysin), 269 (apigenin/galangin), 271 (pinobanksin), 283 (CAPE), 285 (sakuranetin), and 303 (quercetin)* m/z,* have also been reported [1, 27]. These ions were detected in some of the propolis samples in the present investigation.

We also performed PCA with the above loading plots (ions). Figure 2(d) shows the association between abundant ions (213, 217, 219, 229, 245, 249, 261, 263, 265, 279, 295, 311, 313, 314, and 329* m/z*) and the first two PCs. The propolis samples were classified into three groups. Group 1 contained samples 11, 13, 17, 19, 22, and 23; group 2 contained samples 2, 6, 7, 14, 16, 18, and 24; and the rest were classified into group 3 (Figure 2(e)). Figure 2(e) shows that all black propolis samples, except sample 1 (HLJ), were classified into group 1; all yellow propolis samples, except sample 5 (HB-2), belonged to group 2; the greenish-black and yellow-green propolis samples belonged to group 3 (Figure 2(e)). These findings were very consistent with Figure 2(b), indicating a significant correlation between the color of propolis samples and the first two PCs. A recent study also showed a similar relation between propolis color and elemental profiles [33]. Figure 2(f) showed that the climatic zone for samples had no significant correlation with the first two PCs, which also coincided with the result shown in Figure 2(c). Therefore, PCA based on SDAPCI-MS could distinguish Chinese propolis samples of differing colors.

The chemical compositional differences of propolis samples are related to the main sources of resins in the plants visited by the bees [10, 11]. As an example, López et al. characterized red propolis samples from Brazil and Cuba, using direct infusion electrospray ionization mass spectrometry (ESI(−)-MS) and PCA and grouped the samples according to their composition and marker compounds [31]. Our results showed that it was difficult to identify the geographical origins of Chinese propolis by the propolis fingerprint (Figures 2(c) and 2(f)). One possible reason for this is that most propolis samples are quite homogeneous. It has been generally accepted and demonstrated by chemical analysis that the main sources of propolis in Europe and China are the bud exudates of* Populus* and their hybrids [7]. As* Populus* species and their hybrids are widely distributed in China, most Chinese propolis samples are of the poplar type [34]. Therefore, it would be anticipated that Chinese propolis samples would exhibit similar fingerprints by HPLC analysis, both for EEP and for WEP [4, 12, 34], and by SDAPCI-MS analysis, as shown in this study (Supplemental Figures  1 and 2). Accordingly, we were unable to identify the geographical origins of propolis samples used in the present work (Figures 2(c) and 2(f)). The groupings of Chinese propolis via PCA is roughly consistent with their colors (Figures 2(b) and 2(e)), which suggests that not poplar but some other plant sources may control the color of Chinese propolis. It is, therefore, worth investigating the chemical components and the plant sources that control the color of Chinese propolis. We also noted that the most intense signal responses were around 100* m/z* (Table 2; Supplementary Figure  1), different from those signals corresponding to larger molecular weight ions (around 300* m/z*) in the above studies. This finding suggested that the direct SDAPCI-MS method has an advantage in signal acquisition for volatile substances in propolis. Therefore, this could be an understandable cause for different groupings of Chinese propolis samples between this study and previous results [28].

#### 3.2.3. Representative SDAPCI-MS Fingerprints

We obtained SDAPCI-MS fingerprints for propolis samples for groups 1, 2, and 3 in the mass range 70 to 500* m/z* and 200 to 350* m/z* and selected GZ-1, GS, AH, and FJ-2 as being representative, as shown in Supplementary Figures  1  and  2, respectively. For the former, the most abundant ions were 93, 121, and 154* m/z* in group 1; 93, 102, 121, 124, and 151* m/z* in group 2; and 78, 92, 94, 121, 154, and 183* m/z* in group 3. For the latter, the common ions for all samples were 213, 217, 219, 229, 245, 249, 261, 263, 265, 279, 295, 311, 313, 314, and 329* m/z*. Among them, 229, 247, 253, and 313* m/z* were also detected by other MS methods [1, 27]. These fingerprints could be regarded as the references for propolis characterization.

#### 3.2.4. Measurement of Quercetin, Chrysin, and Pinocembrin in Propolis by SDAPCI-MS

**Figure 3:**
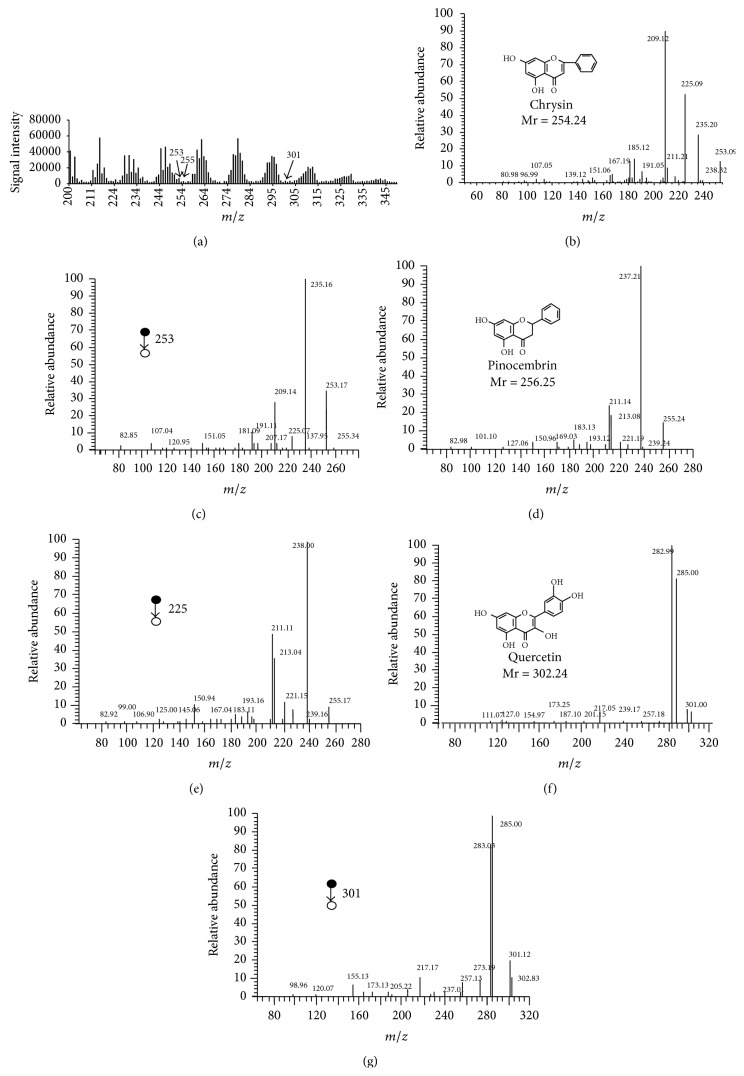
The MS^2^ spectra of propolis. (a) MS spectrum of propolis sample 1 (HLJ) and 253, 255, and 301* m/z* are indicated by arrows. (b), (d), and (f), MS^2^ spectra of reference standards of quercetin (b), chrysin (d), and pinocembrin (f). (c), (e), and (g), MS^2^ spectra of 253* m/z* (c), 255 (e), and 301 (g) in MS spectrum of propolis sample 1 (HLJ).

Most Chinese propolis belongs to the poplar type and is rich in health promoting phenolic compounds [28, 34, 35]. All propolis samples in the present study were analyzed by HPLC. Previous study indicated that 9 chemicals, including chrysin, pinocembrin, and quercetin, were common in Chinese propolis samples, except in the one (named YN-2 in this paper) from Xishuangbanna, a tropical region in Yunnan province [4, 12]. Therefore, chrysin, pinocembrin, and quercetin were detected by reference standards for confirmation [12]. In the SDAPCI mass spectra of 200 to 350* m/z* for the propolis samples, possible signals corresponding to the afore-mentioned chemicals can be noted (Figure 3(a)). Therefore, we undertook further experiments to characterize these signals. Using SDAPCI-MS analysis of the standard solutions, the negative ion ([M–H]^−^) of chrysin (253* m/z*) generated major fragment ions at 235, 225, 209, and 191* m/z* (Figure 3(b)), the negative ion ([M–H]^−^) of pinocembrin (255* m/z*) generated major fragment ions at 237, 221, 213, 211, and 193* m/z* (Figure 3(d)), and the negative ion ([M–H]^−^) of quercetin (301* m/z*) generated major fragment ions at 285, 283, and 217* m/z* (Figure 3(f)). For 253, 255, and 301* m/z* in the SDAPCI-MS fingerprint of crude propolis, similar MS^2^ spectra were obtained to those for the reference standards of chrysin, pinocembrin, and quercetin. Similar results were observed for all propolis samples except YN-2, and the spectra for sample HLJ were shown as an example in Figures 3(c), 3(e), and 3(g). Thus, it was confirmed that chrysin, pinocembrin, and quercetin in crude propolis can be detected by SDAPCI-MS without sample pretreatment.

Furthermore, for the SDAPCI-MS method, crude propolis powder was analyzed directly on a piece of filter paper. Two min were needed for sample preparation together with 3 min on average for direct MS analysis. For other similar methods, such as ESI-MS, EI-MS, and EASI-MS, ethanol extraction was needed before MS analysis [27, 29, 31]. Generally, the time-consuming protocol for ethanol extraction of propolis can take from 1 h to 1 week [27, 29, 31]. Therefore, SDAPCI-MS is recommended for rapid and high-throughput analysis of propolis.

## 4. Conclusion

Using SDAPCI-MS, fingerprints for 24 crude propolis samples in the mass range 70 to 500* m/z* were obtained without sample pretreatment. The samples were classified into three groups by PCA of the MS fingerprints. Group 1 included 6 of 7 black samples, group 2 consisted of 7 out of 8 yellow samples, and group 3 consisted of one black, one yellow, 4 yellow-green, and 5 greenish-black samples. These groupings showed that the colors of the propolis samples had significant correlation with their PCs. Moreover, the classifications seemed not to be related to their geographical origin. These findings were verified by similar PCA results for fingerprints in the range 200 to 350* m/z*. In addition, the fragment ions for chrysin, pinocembrin, and quercetin could be directly detected in crude propolis powders by SDAPCI-MS. Since only 2 min was needed for sample preparation and 3 min for measurement of one sample, SDAPCI-MS fulfills the requirements for rapid high-throughput analysis and characterization of propolis.

## Supplementary Material

Under the optimized experimental conditions, SDAPCI-MS fingerprints of 24 propolis samples were obtained for ranges of *m/z* 70-500 and *m/z* 200-350. According to the classification of PCA (Figure 2), representative fingerprints of four Chinese propolis samples, GZ-1, GS, AH and FJ-2, were exhibited in Supplemental Figure 1 (*m/z* 70-500) and Supplemental Figure 2 (*m/z* 200-350).

## Figures and Tables

**Table 1:** Characteristics of the propolis samples.

Sample number	Sample name	Collected area	Climatic zone	Color
1	HLJ	Heilongjiang	Te^a^	B^g^
2	JL	Jilin	Te	Y^d^
3	NM	Neimenggu	Te	B-^f^
4	HB-1	Hebei	Te	Y-^e^
5	HB-2	Hebei	Te	Y
6	SD	Shandong	Te	Y
7	JS	Jiangsu	Su^b^	Y
8	AH	Anhui	Su	B-
9	FJ-1	Fujian	Su	B-
10	FJ-2	Fujian	Su	Y-
11	GD-1	Guangdong	Su	B
12	GD-2	Guangdong	Su	Y-
13	XJ	Xinjiang	Te	B
14	GS	Gansu	Te	Y
15	NX	Ningxia	Te	B-
16	SX-1	Shaanxi	Te	Y
17	SX-2	Shaanxi	Te	B
18	QH	Qinghai	Te	Y
19	GZ-1	Guizhou	Su	B
20	GZ-2	Guizhou	Su	Y-
21	YN-1	Yunnan	Su	B-
22	SC	Sichuan	Su	B
23	YN-2	Yunnan	Tr^c^	B
24	IL	Illinois, USA	Te	Y

*Note*. The criterion of color was based on the Pantone international color system formula guide-C, North America. ^a^Temperate zone. ^b^Subtropical zone. ^c^Tropical zone. ^d^Yellow (102C). ^e^Yellow-green (1395C). ^f^Greenish-black (405C). ^g^Black (433C).

**Table 2:** The loadings and scores for the first three rotated principal components.

	The loadings				The scores			
	[M–H]^−^	PC1	PC2	PC3	Sample number	PC1	PC2	PC3
A^a^	78	−0.157	−0.441	−0.159	1	**−1.145**	−0.105	−0.262
	92	0.139	−0.311	−0.186	2	**1.555**	**−1.286**	**1.080**
	93	−0.271	**0.556**	0.190	3	**−0.747**	**−0.809**	−0.360
	94	−0.341	−0.205	−0.357	4	−0.065	−0.468	−0.058
	102	−0.199	**0.558**	0.190	5	**−0.519**	**−0.625**	−0.010
	121	**−0.629**	0.134	0.096	6	**1.369**	−0.370	**−3.764**
	124	**0.565**	−0.416	0.128	7	**1.019**	**−0.501**	0.279
	151	−0.049	0.287	**−0.552**	8	**−1.192**	**−0.533**	0.052
	154	−0.338	0.434	0.103	9	−0.002	−0.241	**−0.825**
	183	−0.184	**0.545**	−0.294	10	0.070	−0.344	−0.071

B^b^	253	**0.685**	**0.610**	0.091	11	**−0.985**	**0.717**	−0.212
	255	0.227	**0.629**	−0.207	12	**−0.856**	**−0.588**	0.125
	269	**0.593**	**0.541**	−0.061	13	−0.202	**1.338**	**0.629**
	271	0.372	**0.670**	0.084	14	**1.358**	−0.087	**−0.691**
	283	**0.752**	0.268	−0.132	15	−0.418	−0.347	0.349
	313	**0.947**	0.003	−0.182	16	0.400	0.329	**−0.500**
	247	**0.929**	−0.038	0.023	17	**−0.588**	**0.985**	−0.235
	303	**0.816**	0.482	0.079	18	**0.958**	0.446	**0.682**
	163	0.035	**0.566**	0.050	19	**−1.020**	**1.451**	−0.252
	177	0.259	**0.807**	0.064	20	**−1.202**	−0.801	−0.001
	229	**0.511**	−0.165	0.731	21	−0.433	−0.235	**0.825**
	267	**0.913**	0.221	−0.050	22	**−0.579**	**1.695**	**0.960**
	285	**0.660**	0.314	0.175	23	−0.109	**2.384**	**0.561**
					24	0.437	**−1.205**	**1.700**

Bold font indicates high correlation. ^a^The most abundant ions for SDAPCI-MS fingerprints of samples in this paper; ^b^Some abundant ions for fingerprints of propolis samples by other MS methods (Pietta et al., 2002 [1]; Sawaya et al., 2004 [27]). PC: principal component.

**Table 3:** The loadings and the scores for the first three rotated principal components.

	Loadings				Scores			
	[M–H]^−^	PC1	PC2	PC3	Sample number	PC1	PC2	PC3
A^a^	213	0.369	0.204	**0.881**	1	**−0.815**	**−0.509**	−0.424
	217	0.288	0.313	−0.186	2	**2.601**	0.080	**−1.040**
	219	**0.785**	0.284	**0.505**	3	**−0.517**	**−0.796**	0.465
	229	0.276	−0.082	**0.944**	4	0.322	**−0.553**	0.069
	245	**0.604**	0.214	**0.668**	5	−0.366	−0.416	0.076
	249	**0.948**	0.166	0.208	6	**0.979**	−0.282	**3.289**
	261	**0.774**	0.394	0.385	7	**1.163**	−0.454	**0.784**
	263	**0.914**	0.154	0.255	8	**−0.809**	**−0.796**	**−0.608**
	265	**0.892**	0.259	0.312	9	−0.014	**−0.823**	0.381
	279	**0.912**	0.207	0.269	10	0.011	**−0.542**	**1.410**
	295	**0.861**	0.289	0.35	11	**−1.280**	**0.620**	−0.498
	311	**0.838**	0.331	0.379	12	−0.484	**−0.508**	−0.076
	313	**0.941**	0.207	0.115	13	**−0.806**	**0.929**	**−0.510**
	314	**0.944**	0.254	0.072	14	**0.684**	−0.222	**1.563**
	329	**0.703**	0.164	0.259	15	−0.407	**−0.676**	−0.343

B^b^	253	0.434	**0.678**	0.321	16	**0.864**	0.101	0.168
	255	0.050	**0.760**	−0.096	17	**−0.785**	0.286	−0.419
	269	0.390	**0.652**	0.175	18	**1.384**	−0.364	**−0.742**
	271	0.089	**0.742**	0.284	19	**−1.438**	0.040	**−0.618**
	283	**0.615**	**0.508**	0.154	20	**−0.799**	**−0.866**	−0.424
	247	**0.890**	0.129	0.320	21	0.371	**−0.746**	−0.318
	303	**0.574**	**0.718**	0.317	22	**−0.647**	**0.805**	−0.018
	267	**0.772**	0.446	0.295	23	**−0.675**	**0.732**	−0.481
	285	0.449	0.482	0.414	24	**1.967**	−0.363	**−1.688**

Bold fonts indicate high correlation; ^a^the most abundant ions for SDAPCI-MS fingerprints of samples in this study; ^b^some abundant ions of fingerprints of propolis samples by other MS methods (Pietta et al., [1] 2002; Sawaya et al., 2004 [27]).
