# Supplementary figures and images for: Fast Differential Analysis of Propolis Using Surface Desorption Atmospheric Pressure Chemical Ionization Mass Spectrometry
Source: Int J Anal Chem. 2015 Aug 3;2015:176475. doi: 10.1155/2015/176475 (PMC4539062; doi:10.1155/2015/176475)

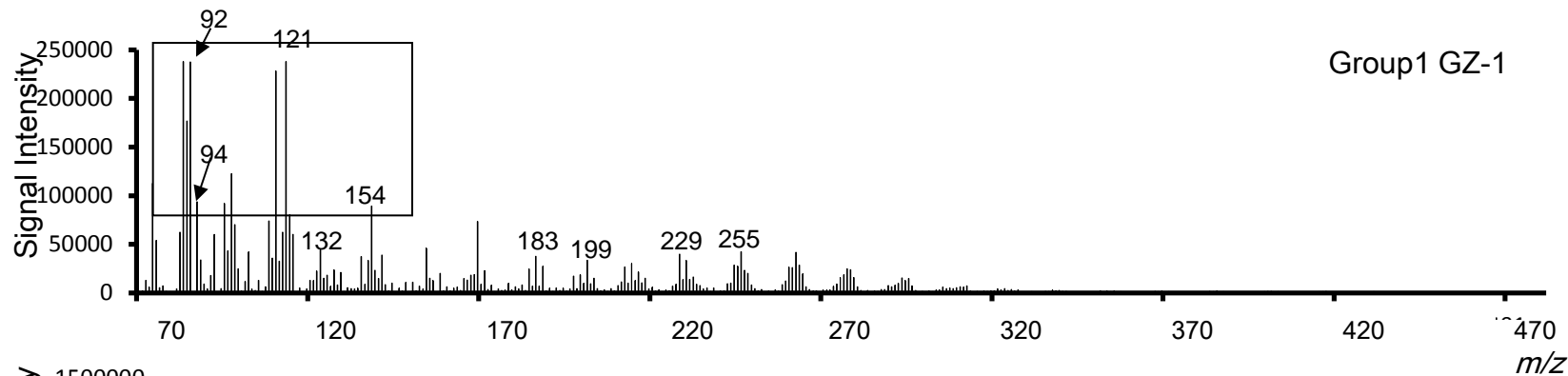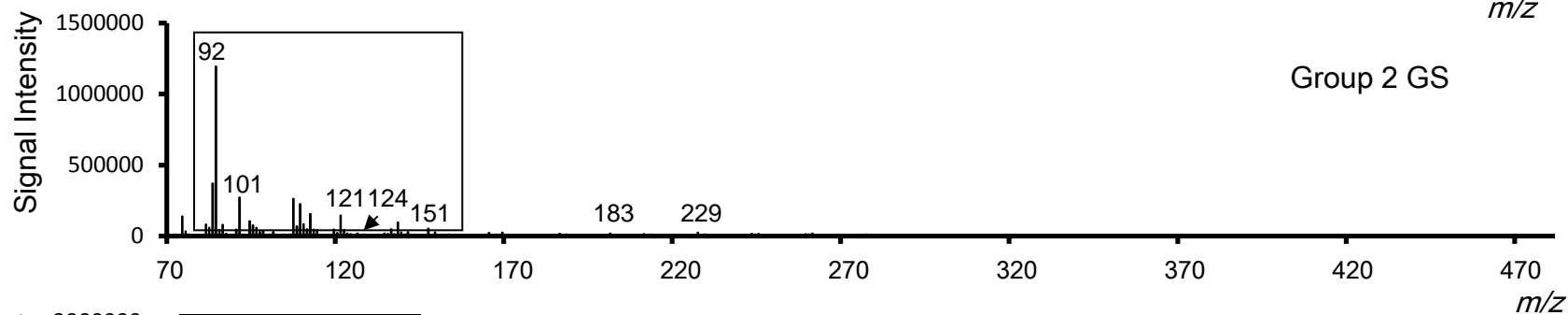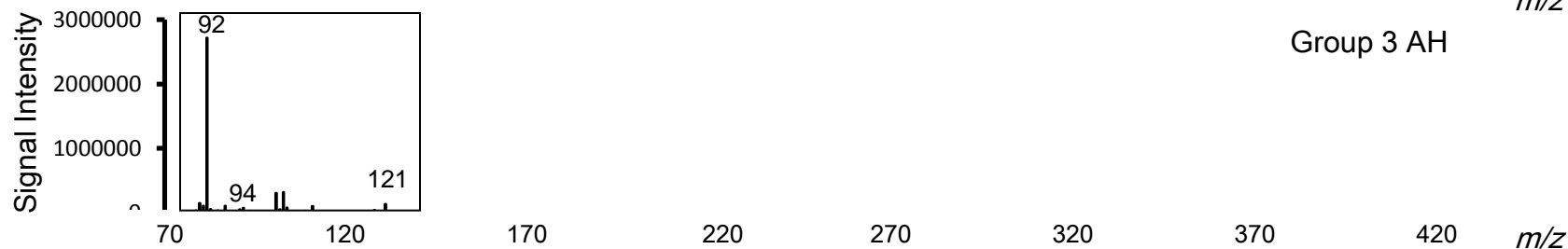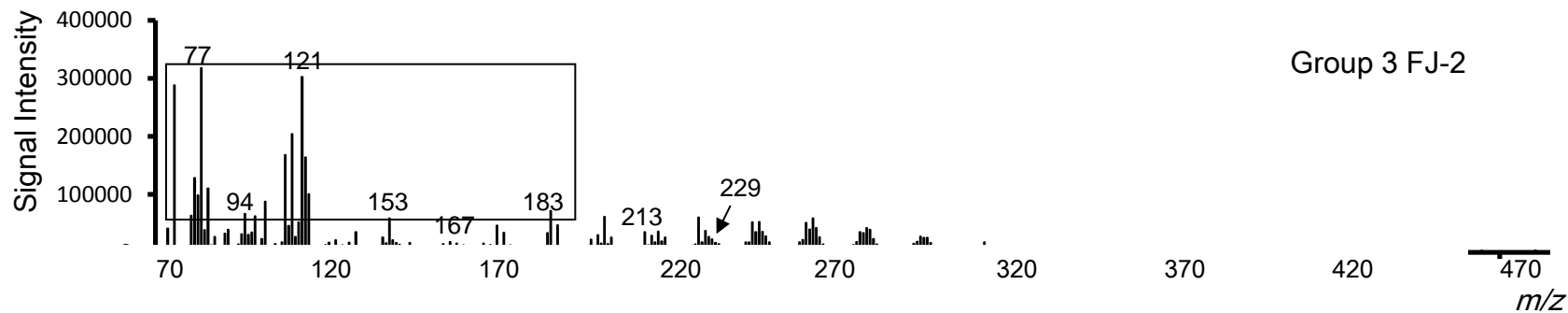

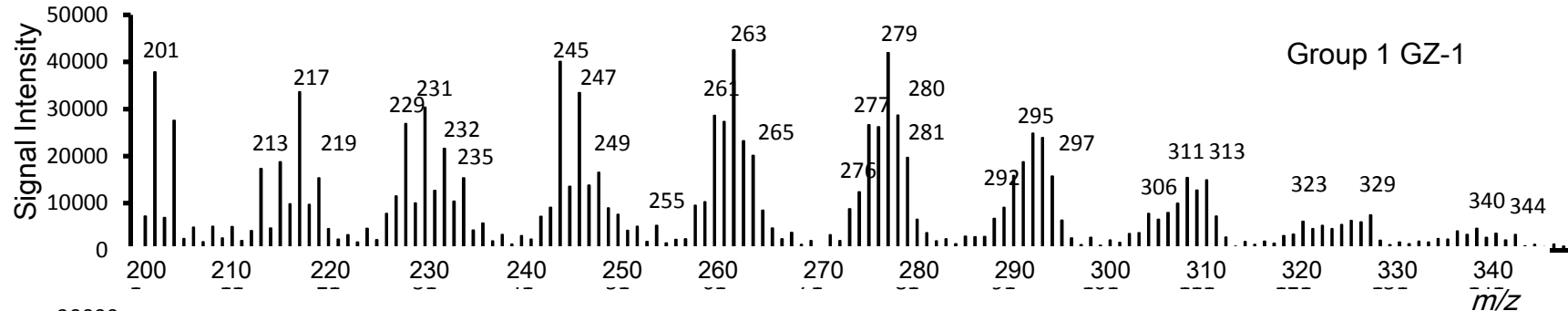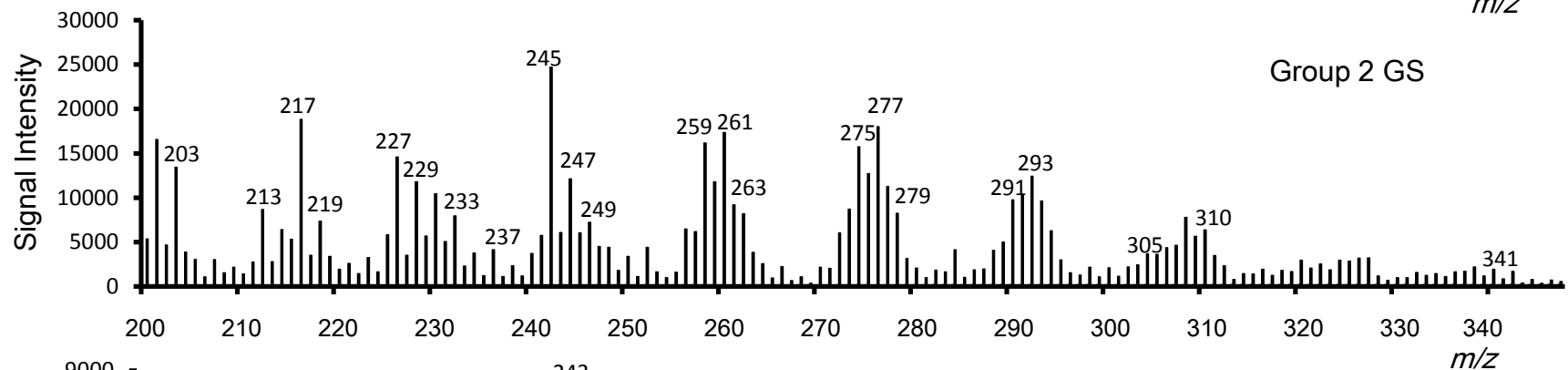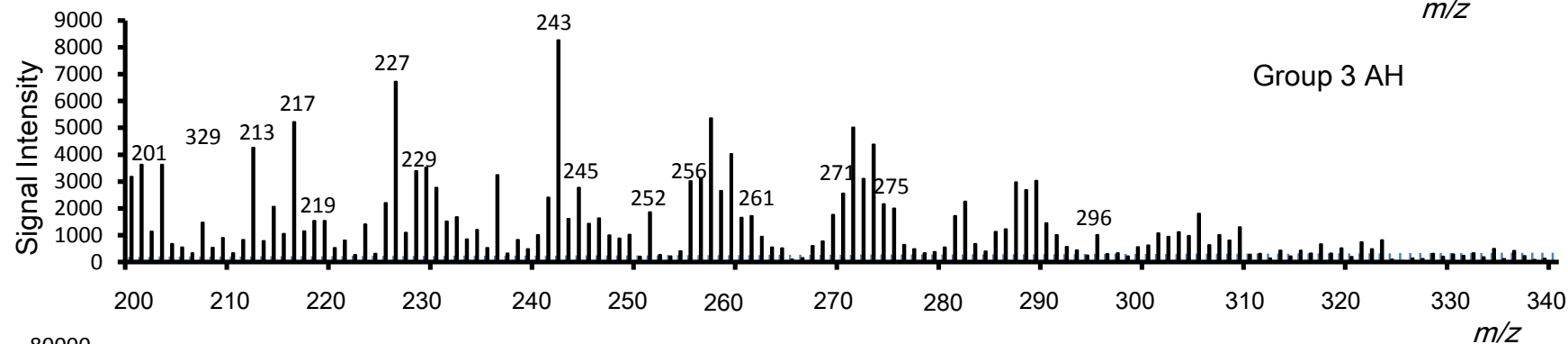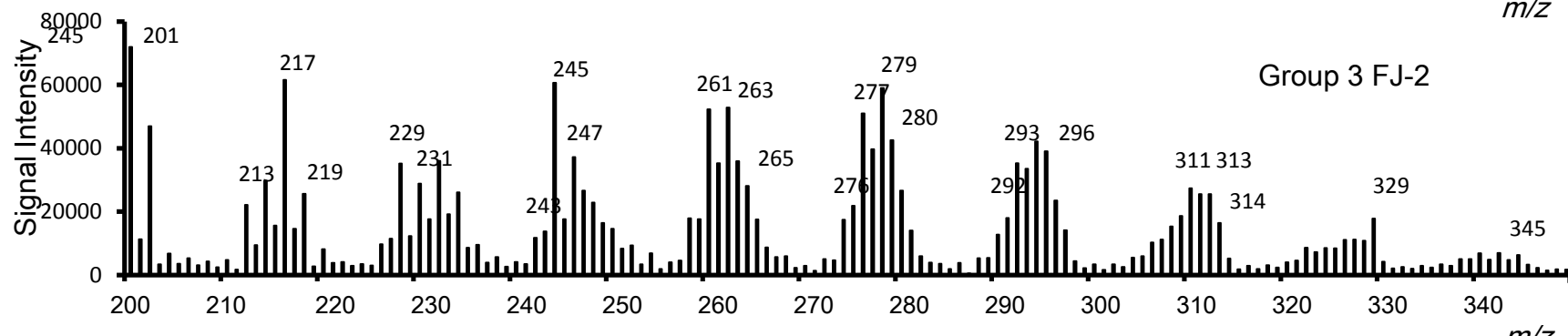

Supplement: Supplementary file 1 — Under the optimized experimental conditions, SDAPCI-MS fingerprints of 24 propolis samples were obtained for ranges of m/z 70-500 and m/z 200-350. According to the classification of PCA (Figure 2), representative fingerprints of four Chinese propolis samples, GZ-1, GS, AH and FJ-2, were exhibited in Supplemental Figure 1 (m/z 70-500) and Supplemental Figure 2 (m/z 200-350). [file 176475.f1.pdf]
